# PPAR*α* Gene Is Involved in Body Composition Variation in Response to an Aerobic Training Program in Overweight/Obese

**DOI:** 10.1155/2021/8880042

**Published:** 2021-08-09

**Authors:** Glêbia A. Cardoso, Mateus D. Ribeiro, Bruno R. V. Sousa, Yohanna de Oliveira, Klécia F. Sena, Joane R. E. Batista, Antônio E. M. Almeida, João M. Filho, Raquel S. B. Silva, Darlene C. Persuhn, Alexandre S. Silva

**Affiliations:** ^1^Laboratory of Applied Studies in Physical Training to Performance and Health-LETFADS, Department of Physical Education, Federal University of Paraíba, João Pessoa, CEP: 58059-900 Paraíba, Brazil; ^2^Associate Graduate Program in Physical Education-UPE/UFPB, Department of Physical Education, Federal University of Paraíba, João Pessoa, CEP: 58059-900 Paraíba, Brazil; ^3^Graduate Program in Nutrition Sciences, Department of Nutrition, Federal University of Paraíba-PPGCN/UFPB, João Pessoa, CEP: 58051-900 Paraíba, Brazil; ^4^Lauro Wanderley University Hospital-HULW-Federal University of Paraíba-UFPB, João Pessoa, CEP: 58059-900 Paraíba, Brazil

## Abstract

The objective of this study was to investigate the relationship of the polymorphism in Intron 7 G/C (rs 4253778) of the peroxisome proliferator-activated receptor alpha (PPAR*α*) gene with the magnitude of changes in the body composition of an overweight and obese population that underwent an aerobic training program. Fifty-eight previously inactive men and women, body mass index (BMI) 31.5 ± 2.8 kg/m^2^, 46.5% (*n* = 27) genotyped as CC genotype and 53.5% (*n* = 31) as CA+AA, underwent a 12-week aerobic training (walking/running). Aerobic capacity (ergospirometry), body composition (DXA), and nutritional assessment were made before and 48 h after the experimental protocol. Two-way ANOVA, chi-square test, and logistic regression were used (*p* < 0.05). Twenty-seven volunteers (46.5%) were identified as CC genotype and 31 (53.5%) as CA+AA genotype. Time-group interaction showed that there was no difference in these between two allele groups. However, differences in distribution of respondents or nonresponders according to allele A were identified for fat mass (*p* ≤ 0.003), percentage fat mass (*p* ≤ 0.002), the waist (*p* ≤ 0.009), abdomen (*p* ≤ 0.000), and hip (*p* ≤ 0.001), this difference being independent for the fat mass. Meanwhile, sex, age, and nutritional management have also been found to be influential factors. It is concluded that the PPAR*α* gene is involved in varying body composition in response to an aerobic training program.

## 1. Introduction

Although obesity is considered a top public contemporary health concern pointing to an increase in its prevalence, as seen from 1999 to 2000 through 2017–2018, US obesity prevalence increased from 30.5% to 42.4% [1], and weight loss is still a big challenge, which can be noticed by the discreet magnitude of weight loss seen in publications on this subject. Systematic reviews and meta-analysis show clinically discrete weight reduction ranging from 1.4 to 2.5 kg of the fat mass in a training program lasting 6 to 12 months [2, 3], in each of the original studies. However, there is an individual variation considerably above these reported averages, as results between -9.5 to +2.6 kg [4], -10.2 to +1.7 kg [5], -11.0 to +4.0 kg fat mass (FM) [6], and -4.4 to +4.9 kg FM [7]. Although this variability is contested by the absence of an adequate comparator sample [8], in a recent published clinical trial [9], while the variation in the experimental group was from -6 to +2.7 kg; in the control group, it was much smaller (-2.4 to +2.5 kg).

Adherence to the training program and nutritional behavior can be considered as intervening factors in individual variability in response to training [10], but these factors have not been statistically analyzed in the data of these studies. Genetic factors may contribute, at least in part, to explain individual differences in the obesogenic process [11, 12]. Among the genes that have been shown to influence body composition, the peroxisome proliferator-activated receptor (PPAR), composed of three subtypes: PPAR*α* involved in glucose metabolism [12], PPAR*γ* related to lipid metabolism [13], and PPAR*σ* more linked to energy balance [14]. In a multicenter trial conducted with 3.234 participants, a variant in PPAR*γ* was positively associated with body mass index (BMI) and visceral adiposity [15]. The same was also demonstrated for the Pro12Ala variant of PPAR*γ* [16]. Besides that, in a genomic association study, which included 307 individuals in the Chinese population, a variant (Thr394Thr) of the PPAR*α* gene was associated with central obesity, and about 43.7% of AG genotyped patients were diabetic [17]. However, none of these studies involved physical training and the slimming process.

In a previous study [18], we found that PPAR*γ*2 did not influence exercise-induced weight loss, despite previous data indicating that this gene is involved in adipocyte regulation, growth, and differentiation [19]. On the other hand, the receptor activated by peroxisome-*α* proliferators (PPAR*α*) is most expressed in tissues such as liver, heart, skeletal muscle, intestinal mucosa, and brown adipose tissue [20]. In addition, PPAR*α* is the most involved of the PPAR family in fatty acid metabolism, and its activation lowers lipid levels [21]. However, also for this gene, its influence on exercise-induced weight loss is not known. Considering the involvement of PPAR*α* in lipid metabolism, we can raise the hypothesis that this gene may specifically be involved in the lipolytic process and, consequently, in weight loss induced by a physical training program. This would be particularly relevant from the point of view of using physical training as a tool to reduce obesity levels.

To elucidate this genetic involvement in exercise-induced weight loss, the objective of this study was to investigate the relationship of the polymorphism in Intron 7 G/C (rs 4253778) of the PPAR*α* gene with the magnitude of changes in the body composition of an overweight and obese population that underwent an aerobic training program aimed at reducing body weight and relating sex, age, and nutritional factors (consumption of proteins, fibers, carbohydrates, and fat in the sixth week of intervention) as possible confounding variables.

## 2. Materials and Methods

This was a clinical trial with 58 adult men and women (33.1 ± 7.6 years) who underwent a 12-week aerobic physical training program. The participants were categorized according to the presence of the A allele, so that the statistical analysis was performed with two trained and genotyped groups (CC, *n* = 27, and CA+AA, *n* = 31). To be eligible, participants had to be adults (ages 20 to 45 years), previously classified as insufficiently active (<150 minutes/week of moderate to severe physical activity) as determined by the International Physical Activity Questionnaire [22]; have a BMI of between 25 and 39.9 kg/m^2^ for at least six months; have not changed more than 2 kg in the last three months; do not smoke or consume alcohol (more than two doses/day); do not use medicine, supplements, or thermogenic substances which alter the metabolism; and do not have any diseases (diabetes, coronary artery disease, or hormonal diseases); for women, they should not be menopausal or present symptoms related to the climacteric period. Those who missed two consecutive weeks or 25% of the physical training program or who began dietary intervention, physical exercise, or medication during the program period, as well as those who were injured, were excluded from the study.

**Figure 1:**
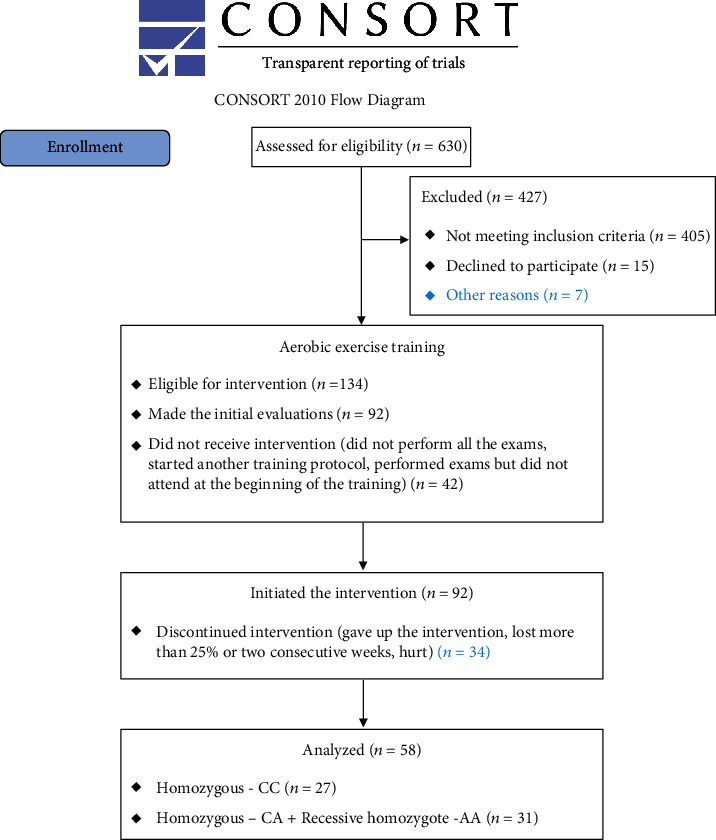
CONSORT flow diagram.

The flowchart in Figure 1 shows the trajectory of recruiting participants from the 630 interested parties who responded to the invitation to participate in the study carried out via social networks until the final samples, considering the study's eligibility and exclusion criteria.

The experimental protocol was approved by the Human Research Ethics Committee of the Health Science Center (CCS) of the Federal University of Paraíba (UFPB), Brazil, under protocol number 1.981.304, and was registered at ClinicalTrials.gov (registration number: NCT03568773). All volunteers who agreed to participate in the study provided written consent after being clarified about procedures and potential risks.

### 2.1. Study Design

**Figure 2:**
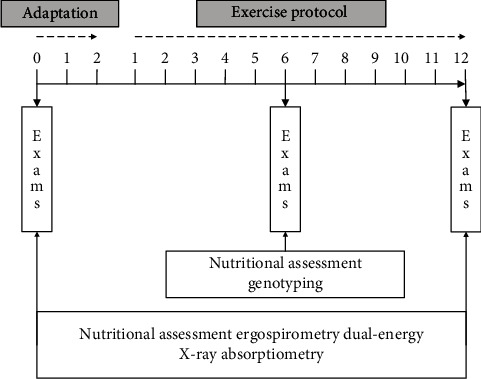
Study design.

Participants were involved in a 12-week consecutive physical training program. Nutritional assessment, ergospirometry and dual-energy X-ray absorptiometry (DXA) were made before the program started, in the 6th week and at the end of the program. Oral mucosa collection was performed for subsequent genotyping (Figure 2).

### 2.2. DNA Extraction and Genotyping

Genomic DNA extracted from the oral epithelial cells were obtained with a 3% sucrose wash of the participants in the experimental group. Extracted DNA was washed with 70% alcohol and resuspended in 40 *μ*L TE buffer (pH 8.0) (25). Polymorphism in Intron 7 G/C (rs 4253778) of the PPAR*α* gene was determined by restriction fragment length polymerase-polymorphism chain reaction (PCR-RFLP), followed by digestion through the restriction enzyme TaqI, generating the following fragments: 266 bp (CC); 266, 216, and 50 bp (CA); and 216 and 50 bp (AA). PCR primers were as follows: the sequence of forward is ACAATCACTCCTTAAATATGGTGG (24 bases); reverse is AAGTAGGGACAGACAGGACCAGTA (24 bases). Cycling conditions were an initial denaturation of 95°C for 2 min, 30 cycles of denaturation 95°C/30 sec, annealing at 56°C/30 sec and extension at 72°C/30 sec, followed by a final extension at 72° C for 5 min.

### 2.3. Nutritional Control

Volunteers underwent a nutritional assessment in order to monitor nutrition through the 24 h recall following a protocol suggested by the Dietary Recommendation Intake [23] behavior throughout the study; it was recommended to maintain the usual dietary patterns. Nutritional assessments were carried out in triplicate, two referring to days of the week and one referring to the weekend. For calculations, AVANUTRI software was used, version 4.0 (Avanutri & Nutrição Computer Services, Três Rios, RJ, Brazil). This assessment was repeated in the sixth and final week.

### 2.4. Body Composition

The volunteer was asked to lie on his back for a full body scanner, using dual-energy X-ray absorptiometry equipment—DXA equipment (LUNAR ADVANCE DF+13.4038 Radiation (GE Lunar Corporation, USA)); the guidelines and procedures of calibration provided by the exams were performed considering the three-compartment model, and its components were divided into the lean tissue, adipose tissue, and bone tissue.

Additionally, circumference measurements were made with a flexible and inextensible measuring tape, with a precision of 1.0 mm (Sanny, São Paulo, Brazil), with the volunteers in an orthostatic position, with the abdomen relaxed, the arms parallel to the body and the feet together, with the tape not compressing the skin and supported parallel to the floor. Waist circumference was measured around the abdomen, taking as reference the average distance between the last floating rib and the iliac crest, abdomen (area with greater abdomen perimeter), and hips (greater gluteal prominence). These measurements took place at the preexperiment moment and 48 hours after intervention. The women were instructed to be in the postmenstrual period before the evaluation.

### 2.5. Aerobic Capacity and Anaerobic Threshold

The ergospirometry test was performed following the individualized ramp protocol [24] with incremental loads at every 3 minutes (Centurion-200 Micromed, Brazil) for determining the maximum VO_2_. The test was performed by a qualified cardiologist. Cardiac monitoring was performed through continuous electrocardiographic tracing (ErgoPC Elite, Micromed, Brazil), always through 13 leads. Blood pressure measurement was performed with a properly calibrated mercury column sphygmomanometer. Exhaled gas was measured using a Metalyzer 3B-Cortex (Leipzig, Germany), which was measured with each breath, associated with the ErgoPC Elite (Micromed, Brazil), and VO_2_ peak was considered as the maximum consumption reached in the last seconds of the exercise. Anaerobic limit (L1) was determined by the agreement of two methods: the V-slope and the ventilatory equivalent. Finally, the respiratory compensation point was determined from the moment of sustained drop in the final expiratory pressure of CO_2_ (PEF CO_2_) and elevation of the expiratory pressure of O_2_ (PEF O_2_). For the interruption of the test, we followed the protocol of [25].

### 2.6. Exercise Protocol

The exercise protocol is shown in Table 1. After two weeks of adaptation with the modality, participants completed 12 consecutive weeks of aerobic training consisting of walking or running. Training prescription was based on the aerobic (L1) and anaerobic (L2) thresholds of each volunteer, according to the results obtained in the ergospirometry test. Initially, the volunteers performed two weeks of adaptation with two sessions/week of 20 to 40 min and intensity below L1 on the treadmill. Then, the training protocol started, from the first to the fourth week; the volunteers performed three sessions/week of 40 to 60 minutes, with intensity at L1. At the fifth week, the frequency and duration of training remained and the intensity was increased to between L1 and 1/2 L2. From the sixth to the 8th week, the training frequency was increased to five sessions/week, with three sessions being held in the laboratory, supervised by the researchers and the application, and two volunteers chose the practice location and did it only with the use of the application for smartphone, to ensure that the training does not leave the zone. From the ninth to the twelfth week, the training frequency and duration remained and the intensity was increased to 1/2 L2 to L2.

The training took place in an open environment during the months of March and April 2018, during which time the city's climate is stable (temperature between 24°C and 30°C throughout the year), with little precipitation (rain). At the time of the sessions (between 6 am and 8 am), the temperature was between 26°C and 28°C with relative humidity of around 80%. These climatic conditions facilitated adherence to the training program and the stress induced by the sessions. All sessions were supervised by an exercise physiologist, and the heart rate was continuously monitored with heart rate monitors (Polar®, model FT1 (Polar Electro Oy, Kempele, Finland)). In addition, each volunteer used a smartphone app to assess distance and training intensity (Endomondo Sports Tracker, version 17.5.1).

### 2.7. Statistical Analysis

Data are presented as mean and standard deviation or the absolute values as variables. Data were tested for normal distribution and homogeneity of variance using Kolmogorov-Smirnov and Levene tests prior to the statistical analyses. Two-way ANOVA for repeated measures (considering the time × allele interaction) to compare differences in body composition variables and nutritional data between the absence (CC) and presence (CA+AA) of allele A. additionally, a chi-square test was used to check possible differences in the distribution of subjects in responders (that reduced the body composition parameters evaluated) and nonresponders (no variation or increase in the selected variables) in the function of the genotype (CC vs. CA+AA). Finally, a binary logistic regression was performed to verify the influence of possible confounding variables on weight loss responses (sex, age, and nutritional factors during the intervention). Data were analyzed using the statistical package SPSS Statistics (v.20, IBM SPSS, Chicago, IL, USA), and the level of significance was set at *p* ≤ 0.05.

## 3. Results

The characteristics of the volunteers in this study are shown in Table 2. From 58 volunteers who completed the training protocol, 70.7% were women (*n* = 41) and 46.5% (*n* = 27) were identified as the CC genotype and 53.5% (*n* = 31) as the CA+AA genotype. The CC group was composed of 10 (37%) subjects overweight and 10 and 17 (63%) obese, and the CA+AA group was composed of 9 (29%) subjects overweight and 22 (71%) obese. When the Hardy-Weinberg Balance was calculated considering *p* ≤ 0.05, we observed that the study sample is consistent with the expected distribution (C = 70% and A = 30%) according to the allele frequency of the European Centre d'Etude du Polymorphisme Humain (45%) reported by the International HapMap Project (http://www.hapmap). When comparing the genotypes, they had similar characteristics in terms of age, level of physical activity compatible with the insufficiently active classification [21], and aerobic capacity between regular and weak according to Brazilian Society of Cardiology [26, 27]. When comparing body composition data, differences were also not found. In addition, the eating pattern was similar.

Time-group interaction analysis is shown in Table 3. Both groups showed significant reductions in the variable's fat mass, percentage fat mass, and circumferences (waist, abdomen, and hip). However, time-group interaction showed that there was no difference in these reductions between two allele groups. For weight and BMI, we observed that only the group with allele A showed an intragroup reduction, but this difference was not maintained in the time-group interaction. As for the lean mass, there was an increase only in the intragroup for CC but also without differences in the time-group interaction. Aerobic capacity showed a significant increase after the intervention, with this improvement occurring in both genotypic groups evaluated, and without differences between the groups.

**Figure 3:**
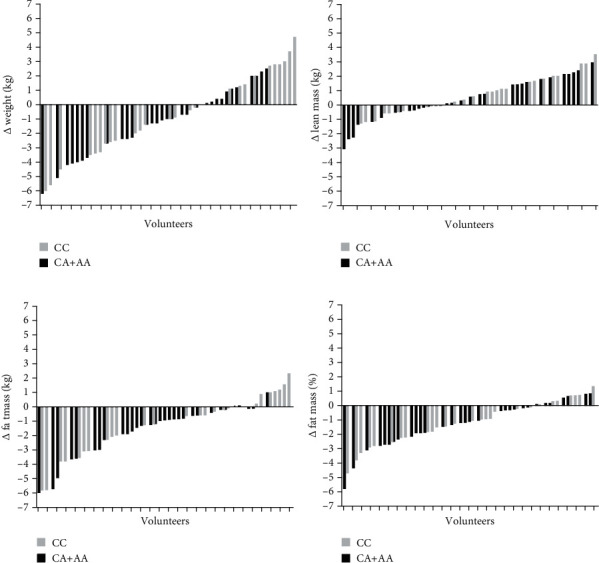
Interindividual difference in the variation of the participants' body composition after intervention. Data are (a) weight, (b) lean mass, (c) fat mass, and (d) fat mass percentage.

Figure 3 shows that there were responders and nonresponders for body composition variables. 38 volunteers (62%) reduced body weight, while 22 volunteers (38%) did not reduce or increase weight. The biggest weight reduction was 6.2 kg, and the biggest increase was 4.7 kg. For percentage fat mass, the biggest increase was 1.4% and the biggest reduction was 5.8%; meanwhile, fat mass had its biggest increase of 2.7 kg and biggest reduction of 5.9 kg. It is worth noting that a variation related to lean mass can also be observed, with volunteers gaining up to 3.6 kg and others reducing up to 3.1 kg.

Although differences in the time × group interaction were not found, when the volunteers were categorized as responders and nonresponders, important differences in responses to training were observed. Among the CC volunteers, it can be seen that 66.7% were respondents for fat mass, while among the CA+AA volunteers, about 90.3% were respondents. Differences can also be noted for the percentage of fat (*p* ≤ 0.002) and circumferences [waist (*p* ≤ 0.009), abdomen (*p* ≤ 0.000), and hip (*p* ≤ 0.001)] (Table 4).

A multiple binary logistic regression to verify sex, age, and nutritional factors (consumption of proteins, fibers, carbohydrates, and fat in the sixth week of intervention) as possible confounding variables was noted; the highest frequency of distribution of the AA allele among subjects who were responsive to reduction of fat mass was independent of these confounding variables, while these variables were shown to influence the percentage of fat mass and circumferences (waist, abdomen, and hip), indicating that genetic influence is not independent (Table 5).

## 4. Discussion

This study showed that the polymorphism in the PPAR*α* gene does not influence the magnitude of weight loss induced by an aerobic training program; however, it showed that the weight loss responsiveness to the training program was genetic-dependent. This dependence was not only genetic because sex, age, and nutritional intake were also influential, but the ability to respond to fat mass was independent of the confounding factors that were considered.

Variability in magnitude of individual responses seen in previous studies was also found in the present study. Meanwhile, some people reduced between 0.07 and 5.99 kg of the fat mass and others increased this measure from 0.03 to 2.70 kg; in previous studies, the results between -9.5 to +2.6 kg [4], -10.2 to +1.7 kg [5], -11.0 to +4.0 kg FM [6], and -4.4 to +4.9 kg FM [7] were observed.

Although the magnitude of weight loss in response to the physical training program has been well demonstrated through the mean, responsiveness to programs is relevant, since it is notorious to identify people who are not able to lose weight after physical training. While our data pointed to an important prevalence of 38% of people who were unresponsive to weight loss, this data has not been presented in previous studies (results presented only as mean and inferential statistics) [8].

Although genetic factors can be easily suggested as possible causes of the variability in slimming responses and responsiveness to training programs, this possibility has so far been scarcely investigated. After mapping the human genome, broad association studies (GWAS) have managed to identify a variety of genes involved in the etiology of obesity, among them MC4R, FTO, and PPAR family genes [28]. However, at least as far as we know, few studies have verified the genetic influence on weight loss, specifically exercise-induced weight loss.

Our laboratory has been trying to identify causal factors for this variability and prevalence of responsiveness. In a previous study, we tested PPAR*γ* and found no influence of this gene on the responses obtained after a continuous aerobic training program [18]. However, PPAR is a family with PPAR*α*, PPAR*γ*, and PPAR*σ*, so that, following this previous study, we decided to check the PPAR*α* gene, since this gene is the most involved of the PPAR family in the fatty acid metabolism, and its activation lowers lipid levels [21]. In fact, this is the first time that it has been demonstrated that some gene influences the occurrence of weight loss induced by a training program, where it was demonstrated that carriers of the allele A have a higher frequency of people who have shown themselves to be responsive. We found evident associations that the genotype influences the frequency of distribution between responders and nonresponders for an aerobic training program in the variables FM, %FM, CC, abdomen, and HC.

Statistical procedures showed this difference in response from the A allele only for the frequency of responsiveness (Table 4), but not in the magnitude of weight loss (Table 3). However, it was noted that despite the lack of difference in the time-group interaction, allele A patients had a greater descriptive response to all weight loss-associated variables. Therefore, we do not rule out that the presence of the PPAR*α* allele A also influences magnitude. We therefore suggest studies with a larger sample size to better test this possibility.

Although our data shows that there is a genetic participation in this process, this participation does not seem to work alone, since most of the associations found (%FM, WC, abdomen, and HC) were not maintained, due to confounding factors such as sex, age, and nutritional factors (consumption of proteins, fibers, carbohydrates, and fat in the sixth week of intervention). In fact, the literature presents some factors that influence the responses found by physical exercise, such as genetic, physiological, environmental, and ethnic factors, in addition to age, training history, level of physical activity, and social formation [29]. Therefore, the data from the present study, while demonstrating consistently the influence of this polymorphism in the responsiveness to weight loss, also corroborate the fact that environmental influence has a determining role in this process [30].

It is known that the activation of PPAR*α* positively regulates the expressions of several enzymes involved in mitochondrial *β*-oxidation and peroxisome and in microsomal *ω*-oxidation, as well as in the transcriptional regulation of genes necessary to maintain redox balance during fatty oxidative acid catabolism [31]. In addition, PPAR*α* is largely related to molecular actions in lipid metabolism and inflammation and is involved not only in glucose and lipid metabolism but also in inflammation modulation pathways [32]. With regard to physical exercise, the regulation of the expression of this gene can increase the oxidative capacity of skeletal muscle in relation to endurance training [33]; thus, this gene is often found in athletes of this modality [34].

Considering that exercise-induced weight loss and its magnitude are already well defined in several previous studies, our data add the information that responsiveness to the training program is something that must be considered and that genetic and environmental factors determine differences in individual responses, even in the face of a sample made homogeneous before the inclusion criteria. Our data provide ways for health professionals to advance in their work, starting from the classic intervention and evaluating the results for a second moment of the intervention, where causal factors or the absence of weight loss can be identified and remedied, when possible, in order to restore the individual weight loss capacity, pointing out that managing nutritional intake and genetic characteristics is an initial path for this second intervention.

## 5. Conclusion

This study showed that people with CC allele in the PPAR*α* gene have less responsiveness to weight loss induced by a physical training program, while people with CA and AA alleles have 26% higher weight loss. However, our data showed that this dependence was not only genetic because sex, age, and nutritional intake were also influential.

## Figures and Tables

**Table 1:** Exercise protocol.

Week	Sessions/week	Time (min)	Intensity
2	2	20–40	<L1
1	3	40	L1
2–3	3	50	L1
4	3	60	L1
5	3	60	L1–1/2 L2
6–8	5	60	L1–1/2 L2
9–12	5	60	1/2 L2–L2

<L1: below anaerobic threshold; L1: anaerobic threshold; L1–1/2 L2: between anaerobic threshold and half the respiratory compensation point; 1/2 L2–L2: half of respiratory compensation point and respiratory compensation point.

**Table 2:** Baseline characteristics of participants according to the 7 G/C (rs 4253778) polymorphism of the PPAR*α* gene.

Variables	Alleles		
	CC	CA+AA	*p* value
N (%)	27 (46.5%)	31 (53.5%)	
Age (years)	31.6 ± 7.4	34.4 ± 7.7	0.14
PA (min/week)	74.2 ± 27.2	71.1 ± 31.3	0.54
VO_2_max (mL/kg min)	28.5 ± 5.7	29.1 ± 6.3	0.69
Weight (kg)	84.6 ± 11.0	85.3 ± 11.7	0.78
BMI (kg/m^2^)	31.3 ± 2.6	31.7 ± 3.1	0.67
LM (kg)	45.0 ± 8.6	45.4 ± 11.4	0.88
FM (kg)	36.2 ± 6.1	37.2 ± 6.1	0.52
% FM	43.7 ± 5.8	44.5 ± 7.6	0.69
WC (cm)	92.5 ± 8.1	94.0 ± 7.8	0.51
Abdomen (cm)	104.3 ± 7.4	106.1 ± 7.5	0.34
HC (cm)	110.9 ± 5.5	110.6 ± 6.6	0.85
*Nutrition*			
Energy (kcal)	1988.8 ± 542.6	1766.1 ± 561.1	0.13
Carbohydrate (g)	262.3 ± 77.0	235.4 ± 80.7	0.20
Lipids (g)	65.5 ± 20.5	55.3 ± 18.4	0.05^∗^
Protein (g)	86.1 ± 38.2	79.6 ± 35.8	0.49
Fibers (g)	14.9 ± 6.2	13.8 ± 6.7	0.53

Data are means ± SD. *N*: number of participants; PA: physical activity; BMI: body mass index; LM: lean mass; FM: fat mass; %FM: fat mass percentage; WC: waist circumference; HC: hip circumference. ^∗^Between group-group differences (one-way ANOVA) (*p* ≤ 0.05).

**Table 3:** Comparison between effect of training on aerobic capacity and body composition of people with allele C versus A.

Variables	CC (*n* = 27)			CA+AA (*n* = 31)		
	Baseline	After	▲	Baseline	After	▲
VO_2_max (mL kg^−1^)	28.5 ± 5.7	33.2 ± 6.9^∗^	4.7 ± 3.9	29.1 ± 6.3	36.5 ± 10.9^∗^	7.3 ± 7.1
Weight (kg)	84.6 ± 11.0	84.0 ± 12.0	−0.6 ± 2.9	85.3 ± 11.7	84.1 ± 11.2^∗^	−1.2 ± 2.3
BMI (kg/m^2^)	31.3 ± 2.6	31.1 ± 2.8	−0.2 ± 1.3	31.7 ± 3.1	31.2 ± 3.2^∗^	−0.5 ± 0.9
LM (kg)	45.0 ± 8.6	45.7 ± 8.6^∗^	+0.7 ± 1.3	45.4 ± 11.4	45.8 ± 11.1	+0.4 ± 1.6
FM (kg)	36.2 ± 6.1	35.1 ± 7.1^∗^	−1.1 ± 2.3	37.2 ± 6.1	35.7 ± 6.7^∗^	−1.5 ± 1.7
%FM (%)	43.7 ± 5.8	42.6 ± 5.8^∗^	−1.1 ± 1.5	44.5 ± 7.6	43.2 ± 8.2^∗^	−1.3 ± 1.5
WC (cm)	92.5 ± 8.1	91.0 ± 8.4^∗^	−1.5 ± 3.5	94.0 ± 7.8	91.7 ± 7.9^∗^	−2.3 ± 2.1
Abdomen (cm)	104.3 ± 7.4	101.5 ± 8.6^∗^	−2.8 ± 3.6	106.1 ± 7.5	101.9 ± 7.7^∗^	−4.2 ± 3.4
HC (cm)	110.9 ± 5.5	108.8 ± 5.6^∗^	−2.8 ± 2.5	110.6 ± 6.6	107.8 ± 6.9^∗^	−2.8 ± 2.5

Data are means ± SD. BMI: body mass index; LM: lean mass; FM: fat mass; %FM: fat mass percentage; WC: waist circumference; HC: hip circumference. ^∗^Intragroup differences vs. baseline (pairwise Student's *t*-test) (*p* < 0.05). Differences in time × group interaction were not found (two-way ANOVA) (*p* ≤ 0.05).

**Table 4:** Distribution test between the polymorphism in Intron 7 G/C (rs 4253778) of the PPAR*α* gene and responders and nonresponders to weight loss training program.

Variables		PPAR*α*		McNemar test	RR (95% IC)
		CC *n* (%)	CA+AA *n* (%)		
△weight				0.150	0.80 (0.27–2.31)
	Responders	16 (59.3)	20 (64.5)		
	Nonresponders	11 (40.7)	11 (35.5)		
△BMI				0.200	0.92 (0.32–2.63)
	Responders	16 (59.3)	19 (61.3)		
	Nonresponders	11 (40.7)	12 (38.7)		
△LM				0.122	1.44 (0.49–4.21)
	Responders	18 (66.7)	18 (33.3)		
	Nonresponders	9 (58.1)	13 (41.9)		
△FM				0.003	0.21 (0.05–0.90)
	Responders	18 (66.7)	28 (90.3)		
	Nonresponders	9 (33.3)	3 (9.7)		
△ %FM				0.002	0.68 (0.19–2.36)
	Responders	20 (74.1)	25 (80.6)		
	Nonresponders	7 (25.9)	6 (19.4)		
△WC				0.009	0.48 (0.14–1.59)
	Responders	18 (66.7)	25 (80.6)		
	Nonresponders	9 (33.3)	6 (19.4)		
△abdomen				0.000	0.84 (0.21–3.30)
	Responders	22 (81.5)	26 (83.9)		
	Nonresponders	5 (18.5)	5 (16.1)		
△HC				0.001	0.42 (0.10–1.64)
	Responders	20 (74.1)	27 (87.1)		
	Nonresponders	7 (25.9)	4 (12.9)		

Data are frequency of responders (who obtained some weight loss) and nonresponders (who did not lose weight or increased any variable related to weight loss). *N*: number of participants; △: variation between pre- and postintervention; BMI: body index mass; FM: fat mass; %FM: fat mass percentage; WC: waist circumference; HC: hip circumference. Chi-square test (McNemar test).

**Table 5:** Logistic regression model verifying the influence of sex, age, and nutritional factors between Intron 7 G/C polymorphism (rs 4253778) and body composition.

	*β*	*p*	RR (95% IC)
FM	−3.226 ± 1.150	0.005	0.04 (0.004–0.378)
%FM	−0.788 ± 0.796	0.322	0.455 (0.095–2.165)
WC	−950 ± 0.719	0.187	0.387 (0.95–1.584)
Abdomen	−0.161 ± 0.824	0.845	0.851 (0.169–4.280)
HC	−1.289 ± 0.896	0.150	0.276 (0.048–1.595)

Dependent variable. △: variation between pre- and postintervention; FM: fat mass; %FM: fat mass percentage; WC: waist circumference; HC: hip circumference. Independent variables: G/C polymorphism, sex, age, and nutritional factors (ingestion carbohydrates, fat, proteins, and fibers in the sixth week of intervention). For each body composition variable, a separate model was made.

## Data Availability

The data of this study are available from the corresponding author upon reasonable request.

## Additional Points

*Highlights*. (i) The polymorphism in the PPAR*α* gene influences the weight loss induced by twelve weeks of physical training with aerobic exercises. (ii) Responsiveness to the training program is something that must be considered, and genetic and environmental factors determine differences in individual responses.
